# Light and Heavy Fractions of Soil Organic Matter in Response to Climate Warming and Increased Precipitation in a Temperate Steppe

**DOI:** 10.1371/journal.pone.0033217

**Published:** 2012-03-30

**Authors:** Bing Song, Shuli Niu, Zhe Zhang, Haijun Yang, Linghao Li, Shiqiang Wan

**Affiliations:** 1 State Key Laboratory of Vegetation and Environmental Change, Institute of Botany, Chinese Academy of Sciences, Xiangshan, Beijing, China; 2 Graduate School of Chinese Academy of Sciences, Yuquanlu, Beijing, China; 3 Key Laboratory of Plant Stress Biology, College of Life Sciences, Henan University, Kaifeng, Henan, China; Argonne National Laboratory, United States of America

## Abstract

Soil is one of the most important carbon (C) and nitrogen (N) pools and plays a crucial role in ecosystem C and N cycling. Climate change profoundly affects soil C and N storage via changing C and N inputs and outputs. However, the influences of climate warming and changing precipitation regime on labile and recalcitrant fractions of soil organic C and N remain unclear. Here, we investigated soil labile and recalcitrant C and N under 6 years' treatments of experimental warming and increased precipitation in a temperate steppe in Northern China. We measured soil light fraction C (LFC) and N (LFN), microbial biomass C (MBC) and N (MBN), dissolved organic C (DOC) and heavy fraction C (HFC) and N (HFN). The results showed that increased precipitation significantly stimulated soil LFC and LFN by 16.1% and 18.5%, respectively, and increased LFC∶HFC ratio and LFN∶HFN ratio, suggesting that increased precipitation transferred more soil organic carbon into the quick-decayed carbon pool. Experimental warming reduced soil labile C (LFC, MBC, and DOC). In contrast, soil heavy fraction C and N, and total C and N were not significantly impacted by increased precipitation or warming. Soil labile C significantly correlated with gross ecosystem productivity, ecosystem respiration and soil respiration, but not with soil moisture and temperature, suggesting that biotic processes rather than abiotic factors determine variations in soil labile C. Our results indicate that certain soil carbon fraction is sensitive to climate change in the temperate steppe, which may in turn impact ecosystem carbon fluxes in response and feedback to climate change.

## Introduction

As atmospheric CO_2_ concentrations are rising, global temperature has increased and will continue to increase in the future [1]. Simultaneously, changes in global and regional precipitation regimes are expected [2], [3]. The climate change could profoundly affect ecosystem carbon (C) and nitrogen (N) cycles, with consequent increase or decrease in soil C and N storage and negative or positive feedback to climate change. Global soils contain 1500 Pg (1Pg = 10^15^ g) of organic carbon in the top soil layer to the depth of 1 m, which is more than the amounts of C in the atmosphere and vegetation combined [4], [5]. Even slight change in the amount of soil C may dramatically influence atmospheric CO_2_ concentration [6]. The stocks of soil C result from the balance between carbon inputs and outputs. Therefore, any factors impacting carbon inputs (net primary productivity) and outputs (dominated by soil respiration) could change the quantity of organic carbon in soils [7].

Soils contain thousands of different organic-C compounds which have mean residence times ranging from years to millennia [7], [8]. Soil organic carbon (SOC) is usually divided into labile C with a small size and rapid turnover and recalcitrant C with a large size but slow turnover. Generally, it is difficult to detect significant changes in soil total C and N contents because of their high background values and great heterogeneity of soils [9]. However, separating soil carbon into different physical or chemical components and then examining their individual responses to climate change is a useful way to detect signals in soil carbon changes [7]. For example, soil labile C was documented to be more sensitive to alterations in moisture, temperature and plant species [10] in comparison with recalcitrant C. So, identifying the fractions of soil organic matter (SOM) into different pools and quantitatively analyzing these pools changes are critical for better understanding C and N dynamics and their responses to climate change.

Previous studies have documented that SOC contents would decrease greatly with global warming [11], [12], [13], and increase [5], [14] or stay constant [15] with precipitation change, but how labile and recalcitrant soil organic C respond to climate change remains unclear. The key issue whether soil labile C and recalcitrant C respond differently to temperature change is still in debate [16], [17], [18]. In particular, there is lack of field experimental evidence on the responses of soil labile and recalcitrant C to climate change [19].

Grassland soil represents an important global C reservoir, and stores as much as 20% of global soil C [5]. Thus, the response of grassland soil C to climate change will be an important component of the global soil C feedback to climate change. Here we designed a field experiment manipulating temperature and precipitation changes in a semiarid steppe, which has been conducted for 6 years since April 2005, to examine the influence of climate change on labile and recalcitrant fractions of soil carbon and nitrogen. Previous studies in this field experiment have shown that the gross ecosystem productivity (GEP) and soil respiration (SR) of this steppe ecosystem were both decreased by warming and enhanced by increased precipitation [20], [21]. However, the reduction in GEP by warming was greater than that in SR, and the stimulation in GEP was greater than that in SR under increased precipitation. Thus, we hypothesize that soil carbon will change due to shifts of the balance between carbon gains and carbon losses. The specific objectives of this study were to evaluate (1) how labile and recalcitrant fractions of soil carbon and nitrogen respond to climate warming and increased precipitation, and (2) what factors or processes determine the variations in soil carbon fractions in the context of climate change.

## Materials and Methods

### Study site

The study site is located in a semiarid temperate steppe in Duolun County (42°02′N, 116°17′E, 1324 m a.s.l.), Inner Mongolia, China. Mean annual precipitation is 382.3 mm with approximately 90% occurring in the growing season from May to October. Mean annual temperature is 2.1°C with monthly mean temperature ranging from −17.5°C in January to 18.9°C in July. Dominant species in this grassland are *Stipa krylovii* Roshev., *Artemisia frigida* Willd, *Potentilla acaulis* L., *Agropyron cristatum* (L.) Gaertn, *Cleistogenes squarrosa* (Trin.) Keng and *Allium bidentatum* Fisch. ex Prokh. [20]. The soil in the study site is a chestnut soil according to the Chinese classification or Haplic Calcisol according to the FAO classification, with 62.75±0.04% sand, 20.30±0.01% silt and 16.95±0.01% clay. Soil bulk density and pH are 1.31±0.02 g cm^−3^ and 7.34, respectively.

### Experimental design

The experiment has received the permit for the field study from the land owner, Institute of Botany, the Chinese Academy of Sciences. The experiment used a nested design, with increased precipitation manipulated at the plot level and warming manipulated at the subplot level. There were three blocks with a 44×28 m area. In each block, there were two 10×15 m plots. One plot was assigned as the increased precipitation treatment and the other as the control. Six sprinklers were evenly arranged into two rows in each of the increased precipitation treatment plots. In July and August, 15 mm of water was added weekly to the increased precipitation treatment plots. Thus, a total of 120 mm precipitation was supplied each year.

Within each 10×15 m plot, four 3×4 m subplots with two warmed subplots and two control subplots were arranged randomly. In the warmed subplot, a 1.65×0.15 m MSR-2420 infrared radiant heater (Kalglo Electronics Inc., Bethlehem, PA, USA) that was suspended 2.5 m above the ground had heated the subplot continuously since April 28, 2005. A previous study by Niu et al. has documented that experimental warming elevated soil temperature at 10 cm depth by 1.17°C [22]. In the control subplot, a “dummy” heater with the same shape and size as the infrared radiator was suspended at the same height to simulate the shading effect of the heater. Therefore, the experimental design consisted of 24 subplots with six replicates for four treatments (control, warming, increased precipitation, and warming plus increased precipitation).

### Soil sampling and measurements

Soil samples were collected from the topsoil (0–10 cm) of all the 24 subplots on August 29, 2010. Two soil cores (6 cm in diameter and 10 cm in depth) were taken from each subplot, and then completely mixed to one fresh sample. Each soil sample was divided into two parts after sieving by a 2 mm mesh and removing any visible plant materials. One part of each sample was stored in iceboxes and transported to the laboratory for microbial analysis, and the other part was air-dried for chemical analysis.

Soil microbial biomass C (MBC) and N (MBN) were determined using the chloroform fumigation-extraction method [23]. Briefly, fresh soil samples were adjusted to 60–70% of field water-holding capacity and incubated for 1 week at 25°C. After that parts of each moist soil sample (30 g) were fumigated for 24 h by ethanol-free CHCl_3_. The remainders (30 g) were used as non-fumigated controls. Both the fumigated and non-fumigated samples were extracted with 75 ml of 0.5 M K_2_SO_4_ for 30 min on a shaker. The extracts were filtered through 0.45 µm filters and determined for extracted C by potassium dichromate-bitriol oxidation method [23] and N by Kjeldahl digestion [24]. MBC and MBN were calculated from the differences between extracted C and N contents in the fumigated and non-fumigated samples using conversion factors of 0.38 [25] and 0.45 [24], respectively. And the extracted C in non-fumigated samples was considered as soil dissolved organic C (DOC).

Soil total C and N were measured by a CHNOS elemental analyzer (vario El III, Elementar Analysensysteme GmbH, Hanau, Germany) after the air-dried samples were ground finely.

In this study, we separated soil labile and recalcitrant fractions of SOM by density fractionation which is one of physical fractionation methods used widely [26]. The light fractions with low density (<1.7 g cm^−3^) are partly decayed plant and animal products, while heavy fractions with high density (>1.7 g cm^−3^) referred to humic substance which are generally mineral associated [27], [28]. Specifically, 15 g air-dried soil was placed in a centrifuge tube and added 50 ml of NaI solution with a density of 1.7 g cm^−3^. The tubes were shaken on a shaker for 30 min, and then centrifuged at 3000 rpm for 10 min. The floating light fraction was sucked on a fiberglass filter in a Büchner funnel. This process was repeated twice in order to separate the light and heavy fractions totally. After that, the material remaining at the bottom of the tube (the heavy fraction) was added 50 ml of deionized water, shaken and centrifuged for three times to wash. The light fraction was washed with 50 ml of 0.01 M CaCl_2_ and then 50 ml of deionized water. Both the light fraction and heavy fraction were dried at 60°C for 48 h, weighed and ground to determine the C and N contents using a CHNOS elemental analyzer (vario El III, Elementar Analysensysteme GmbH, Hanau, Germany).

### Statistical analysis

Three-way ANOVA for a blocked nested design was used to test the effects of block, warming and increased precipitation on all measured variables. Linear regression analyses were used to evaluate relationships between soil labile fractions (light fraction C and N, microbial biomass C and N, and dissolved organic C) and gross ecosystem productivity (GEP), ecosystem respiration (ER) and soil respiration (SR). The value of GEP, ER or SR was the yearly mean value from 2005 to 2009. The effects were considered to be significantly different if *p*<0.05. All statistical analyses were conducted with SAS V.8.1 software (SAS Institute Inc., Cary, NC, USA).

## Results

### Total C and N in soil

**Figure 1:**
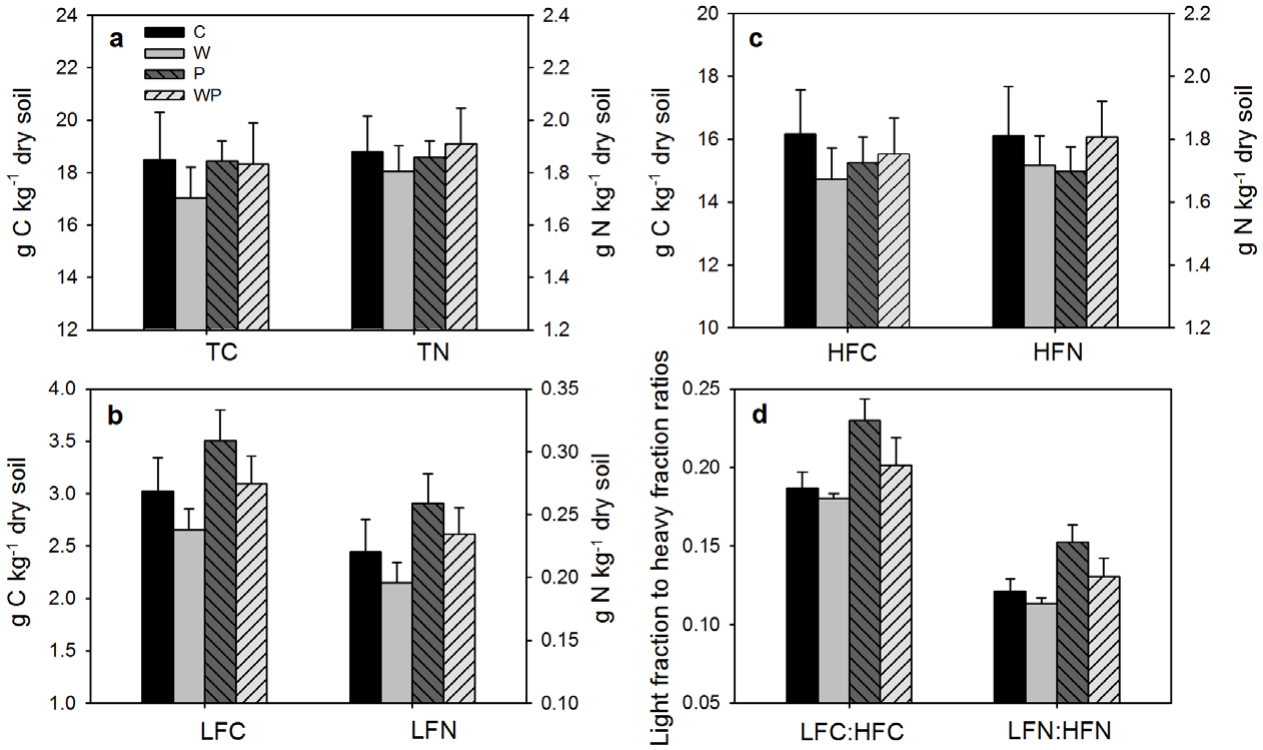
Effects of warming and increased precipitation on soil total C and N (TC, TN) (a), light fraction C and N (LFC, LFN) (b), heavy fraction C and N (HFC, HFN) (c), LFC∶HFC ratio and LFN∶HFN ratio (d) (means ± SE). C, control; W, warming; P, increased precipitation; WP, warming plus increased precipitation.

Soil total C content (TC) was 18.48±1.82 g C kg^−1^ dry soil and total N content (TN) was 1.88±0.14 g N kg^−1^ dry soil (Fig. 1a). Neither warming nor increased precipitation had significant effects on TC or TN after six years of treatments (Table 1, Fig. 1a). The interactive effects of warming and increased precipitation on TC and TN were also not statistically significant (Table 1).

**Table 1:** Results (F and *p* values) of three-way ANOVA on the effects of block (B), warming (W), increased precipitation (P) and their interaction on measured soil variables.

		TC		TN		LFC		LFN		HFC		HFN		LFC∶HFC		LFN∶HFN	
	df	F	*p*	F	*p*	F	*p*	F	*p*	F	*p*	F	*p*	F	*p*	F	*p*
B	2	60.31	**<0.001**	56.13	**<0.001**	7.94	**0.006**	7.44	**0.008**	74.00	**<0.001**	77.54	**<0.001**	0.58	0.573	0.87	0.442
P	1	1.74	0.211	1.23	0.289	5.37	**0.039**	5.51	**0.037**	0.03	0.875	0.11	0.741	5.81	**0.033**	6.39	**0.027**
W	1	2.78	0.121	0.11	0.745	3.93	0.071	2.23	0.161	2.51	0.139	0.05	0.830	1.78	0.207	2.39	0.148
P×W	1	2.05	0.178	2.80	0.120	0.01	0.917	0.00	0.996	5.48	**0.037**	7.67	**0.017**	0.65	0.435	0.52	0.484

TC, soil total C; TN, soil total N; LFC, light fraction C; LFN, light fraction N; HFC, heavy fraction C; HFN, heavy faction N.

### Light and heavy fraction C and N

Heavy fraction C (HFC) and N (HFN) accounted for 84.3% and 89.2% of TC and TN, respectively, in the control plots (Fig. 1). Increased precipitation significantly increased light fraction C (LFC) and N (LFN) by 16.1% and 18.5%, respectively (Table 1, Fig. 1b). The overall warming effects were marginally significant on LFC (*p* = 0.07, Table 1) but insignificant on LFN (*p* = 0.16, Table 1). For example, LFC changed from 3.03±0.32 g C kg^−1^ dry soil in the control plots to 2.66±0.20 g C kg^−1^ dry soil in the warmed plots with ambient precipitation. The interactions between warming and increased precipitation had no significant impacts on LFC and LFN. Neither HFC nor HFN were changed by warming or increased precipitation (Table 1, Fig. 1c).

The ratio of LFC to HFC (LFC∶HFC) was significantly enhanced from 0.18 in the control plots to 0.23 in the increased precipitation plots across both warmed and unwarmed plots. Similarly, ratio of LFN to HFN (LFN∶HFN) was enhanced from 0.12 in the control plots to 0.15 in the increased precipitation plots (Table 1, Fig. 1d).

### Soil C to N ratios

**Figure 2:**
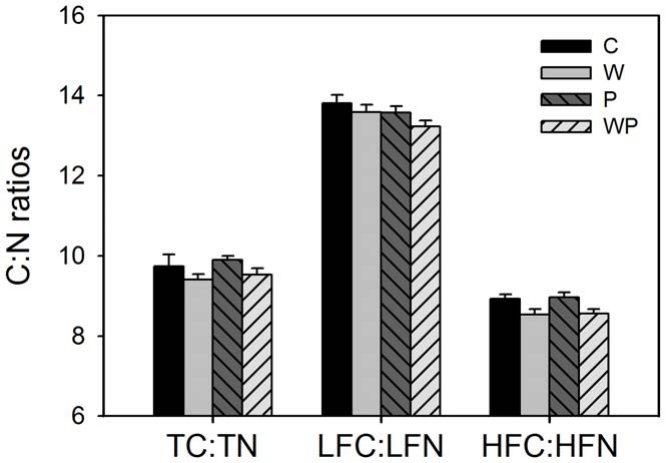
Effects of warming and increased precipitation on ratios of soil C∶N (TC∶TN), light fraction C∶N (LFC∶LFN) and heavy fraction C∶N (HFC∶HFN) (mean ± SE). See Fig. 1 for abbreviations.

Total soil C∶N ratio (TC∶TN) was 9.74±0.29 in the control plots (Fig. 2). Warming significantly decreased TC∶TN ratio from 9.90±0.10 in the control plots to 9.40±0.14 in the warmed plots across both ambient and increased precipitation treatments. C∶N ratio of heavy fraction (HFC∶HFN) was also decreased from 8.96±0.13 to 8.54±0.13 by warming (Table 2, Fig. 2). Nevertheless, warming did not significantly change C∶N ratio of light fraction (LFC∶LFN) which was 13.81±0.20 in the control plots (Table 2, Fig. 2). Increased precipitation or its interaction with experimental warming had no impacts on any of these variables (Table 2).

**Table 2:** Results (F and *p* values) of three-way ANOVA on the effects of block (B), warming (W), increased precipitation (P) and their interaction on soil C∶N ratios, MBC, MBN and DOC.

		TC∶TN		LFC∶LFN		HFC∶HFN		MBC		MBN		DOC	
	df	F	*p*	F	*p*	F	*p*	F	*p*	F	*p*	F	*p*
B	2	18.15	**<0.001**	1.12	0.359	6.80	**0.011**	0.33	0.725	0.63	0.549	7.83	**0.007**
P	1	1.92	0.191	2.69	0.127	0.11	0.751	1.60	0.230	4.68	0.051	0.05	0.835
W	1	11.12	**0.006**	2.62	0.131	16.67	**0.002**	5.81	**0.033**	1.76	0.210	6.27	**0.028**
P×W	1	0.01	0.917	0.13	0.723	0.00	0.947	0.19	0.667	0.18	0.676	3.01	0.109

MBC, soil microbial biomass C; MBN, soil microbial biomass N; DOC, soil dissolved organic C. See Table 1 for abbreviations of TC, TN, LFC, LFN, HFC, and HFN.

### Soil microbial biomass C and N and dissolved organic C

**Figure 3:**
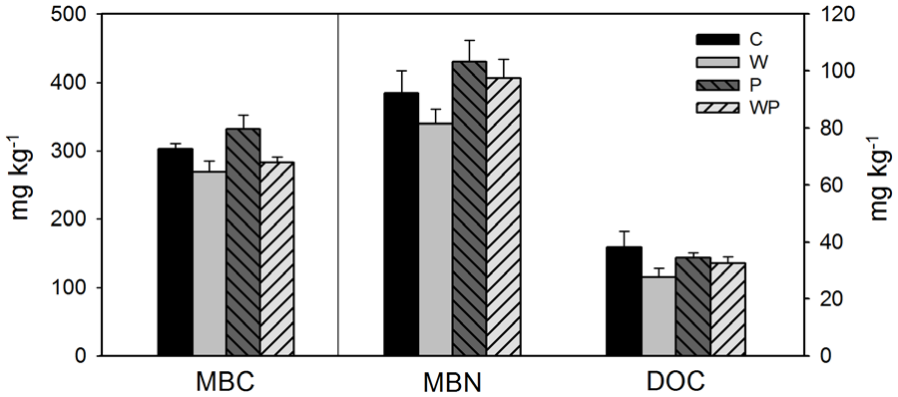
Effects of warming and increased precipitation on soil microbial biomass C and N (MBC, MBN) and soil dissolved organic C (DOC) (means ± SE). See Fig. 1 for abbreviations.

The main effects of warming significantly reduced soil microbial biomass C (MBC) by 12.6% (Table 2, Fig. 3). However, there were no effects of increased precipitation or its interaction with warming on MBC. Soil microbial biomass N (MBN) was not changed by any treatments (Table 2, Fig. 3).

Soil dissolved organic C was decreased from 38.08±5.75 mg kg^−1^ in the control plots to 27.78±2.92 mg kg^−1^ in the warmed plots across the ambient and increased precipitation treatments (Table 2, Fig. 3). Neither increased precipitation nor its interaction with warming had significant effects on DOC (Table 2, Fig. 3).

### Relationships between carbon fluxes and soil C or N fractions

**Figure 4:**
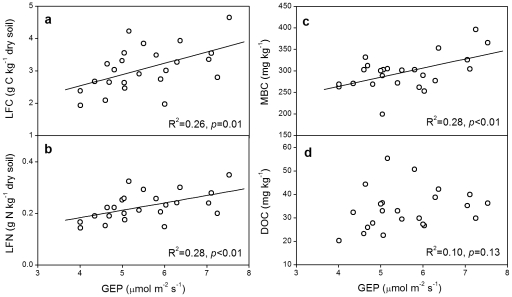
Linear correlations between GEP and light fraction C (a) or N (b), MBC (c) and DOC (d) across all the 24 subplots. GEP, gross ecosystem productivity, whose values were the yearly mean values from 2005 to 2009.

**Figure 5:**
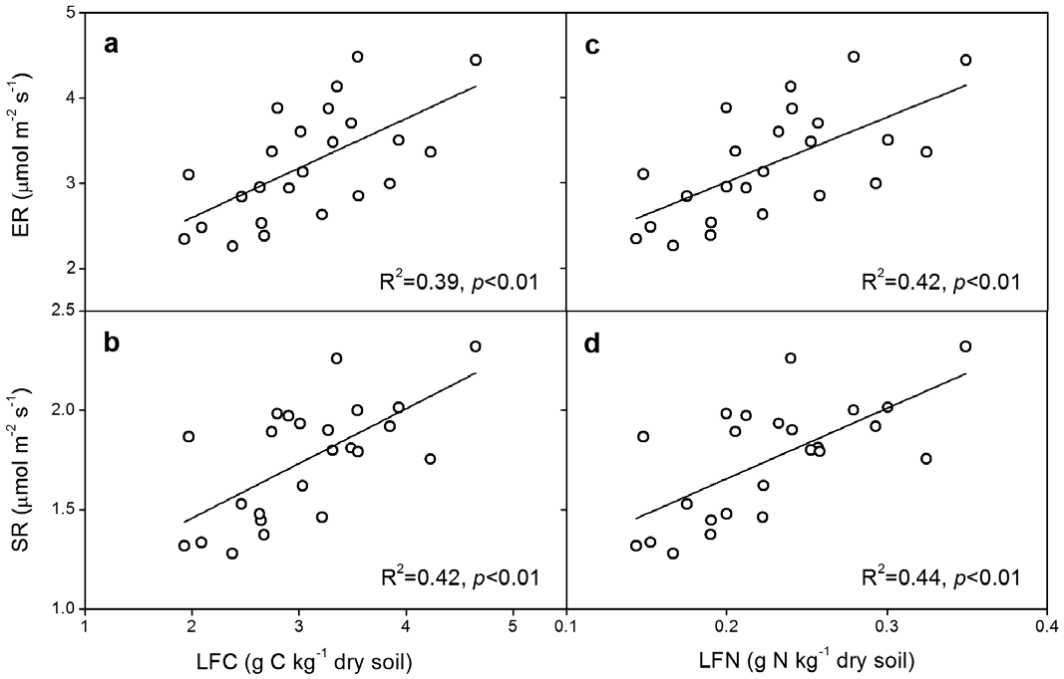
Linear correlations between carbon flux (ER or SR) and light fraction C (a, b) or N (c, d) across all the 24 subplots. ER, ecosystem respiration; SR, soil respiration. The values of ER and SR were the yearly mean values from 2005 to 2009.

**Figure 6:**
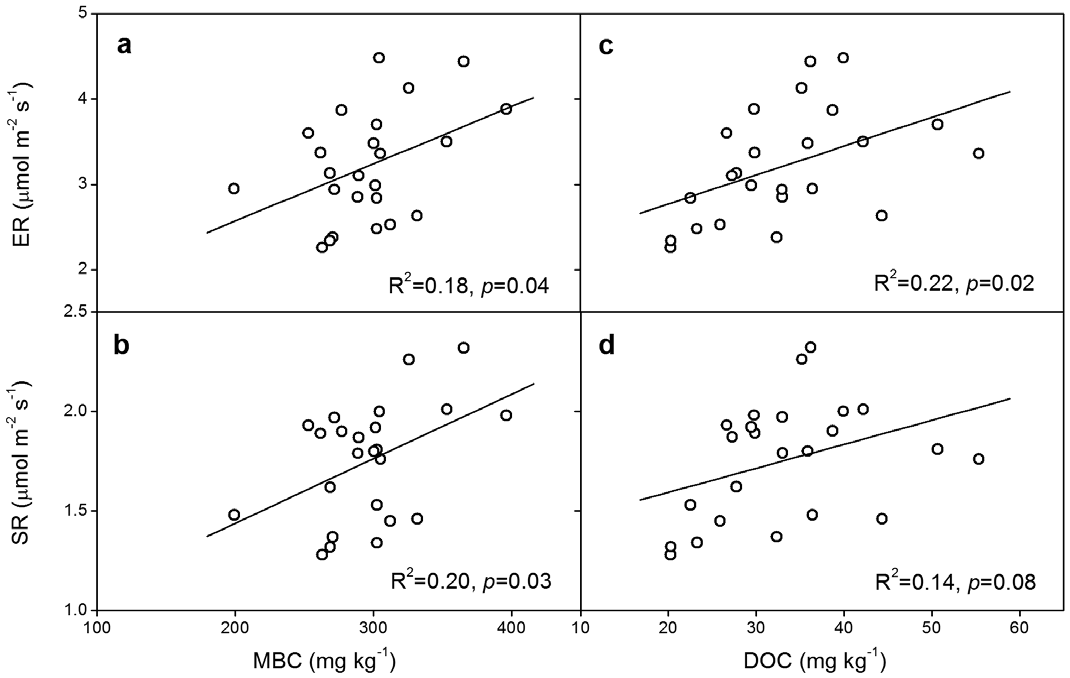
Linear correlations between carbon flux (ER or SR) and MBC (a, b) or DOC (c, d) across all the 24 subplots. ER, ecosystem respiration; SR, soil respiration; MBC, microbial biomass C; DOC, soil dissolved organic C. The values of ER and SR were the yearly mean values from 2005 to 2009.

Across all the 24 subplots, LFC showed a positive relationship with the yearly mean values of GEP (R^2^ = 0.26, *p* = 0.01; Fig. 4a), ER (R^2^ = 0.39, *p*<0.01; Fig. 5a) or SR (R^2^ = 0.42, *p*<0.01; Fig. 5b). Similarly, LFN showed a positive linear correlation with GEP (R^2^ = 0.28, *p*<0.01; Fig. 4b), ER (R^2^ = 0.42, *p*<0.01; Fig. 5c) or SR (R^2^ = 0.44, *p*<0.01; Fig. 5d). Moreover, MBC and DOC, also showed positive linear correlations with GEP, ER and SR, except that DOC had no significant correlation with GEP (*p*>0.05; Fig. 4, Fig. 6). No significant relationship of soil light or heavy fraction C or N was found with soil temperature or moisture across the 24 subplots (*p*>0.05).

## Discussion

### Positive effects of increased precipitation on soil light fraction

Although density fractionation has some uncertainties, such as black carbon issue [26], [29] and potential deficiency in operation [29], it has been widely used for more than 50 years and is well documented to be an effective way for assessing light and heavy pools of SOM that are differently sensitive to environmental changes [29]. As predicted, increased precipitation enhanced soil light fraction C and N in this study (Fig. 1b). The light fraction is a short-term reservoir of plant nutrients and the primary fraction for soil carbon formation, and serves as a readily decomposable substrate for soil microorganisms [10], [30]. Its size is a balance between residue inputs and decomposition [31]. Increased precipitation could stimulate plant growth, leading to more carbon inputs to soil. On the other hand, increased precipitation may directly enhance soil microbial activities and accelerate soil carbon decomposition, inducing more carbon losses from soil. Although increased precipitation could indirectly suppress plant growth and soil microbial activities via reducing soil temperature, in this temperate steppe where water availability plays a dominant role, the positive effects of increased precipitation were much stronger than the indirectly negative effects via reducing soil temperature [21]. In this study, light fraction showed a positive linear correlation with gross ecosystem production (GEP) (Fig. 4) and soil respiration (SR) (Fig. 5). Though both GEP and SR were enhanced by increased precipitation, the stimulation of GEP was greater than that of SR [20], [21]. In addition, plant root production was also improved by increased precipitation [32]. These imply that increased precipitation has resulted in greater substrate inputs to soil than carbon outputs from soil, leading to the positive effects of increased precipitation on soil light fraction C and N.

Soil microbial biomass carbon (MBC) and dissolved organic carbon (DOC) are vital components of ecosystem carbon cycling, which have relatively rapid turnover rate and sever as a source or a sink of labile nutrients. In our study, MBC and DOC were not changed by increased precipitation (Fig. 3), which is consistent with some previous studies [33], but not in accordance with some others [21], [34], [35], [36]. Since microbial biomass and activity are sensitive to changes in soil microenvironment [37], [38], the responses of MBC to increased precipitation can be rapid but short-lived [39]. As we sampled soil in late August when water addition treatment was over, the response of MBC to increased precipitation would not be detected. Another possible reason is that soil temperature in the increased precipitation plots (18.60°C) was markedly lower than that in the control plots (22.65°C) in late August, 2010. Lower soil temperature would constrain microbial activity and growth, which partly compensate the directly positive effect of increased precipitation, leading to little change in MBC.

### Negative effects of experimental warming on soil light fraction

The finding that soil light fraction C was decreased by experimental warming is in accordance with a previous study, in which soil labile C and N were reduced by warming in two forest ecosystems [40]. However, another experiment conducted in a tallgrass prairie showed that experimental warming increased soil labile C and N contents [19]. The discrepancy between our result and the above-mentioned result could be explained by the different controlling factors of C fluxes in different ecosystems. In the tallgrass prairie ecosystem where water is not as limited as in our arid ecosystem, experimental warming could directly stimulate plant growth and microbial activity. The enhancement of above- and below-ground biomass by warming was greater than the stimulation of soil respiration [41], so labile C and N fractions were increased as a result of higher substrate inputs in the tallgrass prairie. However, in the semiarid ecosystem where water availability is the predominate limiting factor [20], warming can exacerbate the dry condition. The negatively indirect warming effect by reducing soil moisture is much stronger than the positively direct effect of improving temperature on ecosystem C fluxes. Previous studies conducted in the same experiment have showed that GEP, ecosystem respiration (ER) [20], SR [21], and plant root production [32] were all reduced by warming. Because light fraction C or N depends on both GEP (Fig. 4) and ER (Fig. 5), more reductions in GEP than those in ER and SR [21] leads to the decrease of light fraction C in soil. There were similar impacts on soil microbial biomass C (MBC) and soil dissolved organic C (DOC). The positive linear relationships between soil MBC and DOC with GEP, ER and SR (Fig. 4, Fig. 6) suggest that the decreases in MBC and DOC are partly due to the decrease in substrate inputs under warming (Fig. 4). This is consistent with a previous study which documented that DOC was positively related to the amount of organic matter inputs [42]. So, we conclude that greater reductions in C gains relative to C losses under climate warming decreased soil light fraction C, MBC and DOC.

Warming decreased total soil C∶N ratio and the C∶N ratio of heavy fraction (Fig. 2), which suggests that soil heavy fraction C has the potential to be decomposed more under warming than in control. Previous studies have documented that soil microbial community structure will change under warming and that the microorganisms preferring more recalcitrant carbon could establish as temperature increases [43]. This means that soil heavy fraction carbon could be preferentially respired by microbes under warming. The decreases of total soil C∶N ratio and heavy fraction C∶N ratio under experimental warming implies that, as global temperature increases, soil heavy fraction C which constitutes the majority of soil carbon may potentially induce increasing C emissions from soil to the atmosphere.

Although it is assumed that abiotic factors associated with climate change may interact to affect ecosystem carbon cycling, there were no significant interactive effects between warming and increased precipitation on soil light fractions of C and N. This is probably due to that 30% increase of precipitation is not enough to alleviate water limitation and to change the negative warming effects. The insignificant interactions between warming and increased precipitation were also reported in previous studies on soil respiration [44], [45] and above-ground biomass production [46].

### Changes in soil total and heavy fraction C and N

Because of the large pool size, significant change in soil total C content in response to climate change is usually difficult to detect in a short time. In the present study, we did not detect significant changes in soil total C or N contents even after 6 years' treatment of experimental warming or increased precipitation (Fig. 1a). Soil heavy fraction C and N, which account for approximately 85% of total C or N, were not affected by either experimental warming or increased precipitation (Fig. 1c). The results are consistent with previous studies which found that soil mineral C did not change after 13 years of increased rainfall [47] and that soil recalcitrant C were not influenced by experimental warming [19], [40]. These indicate that soil heavy fraction C is relative stable to climate change [16], [48].

In conclusion, although soil total carbon and heavy fraction carbon were not affected by increased precipitation or warming, soil light fraction carbon was significantly increased by water addition and decreased by experimental warming after 6 years of treatments in a semiarid temperate steppe. The changes in soil labile C and N were primarily due to the different responses of carbon uptake and release rather than the changes in environmental conditions under treatments. The sensitive responses of soil light fraction C and N, microbial biomass C, and dissolved organic C to climate change indicate that climate warming and increased precipitation may impact carbon cycling by changing certain fractions of soil organic matter. Models should take into account of the fractions of soil organic matter to more accurately predict ecosystem's response and feedback to climate change.
